# Membrane Adsorber for the Fast Purification of a Monoclonal Antibody Using Protein A Chromatography

**DOI:** 10.3390/membranes9120159

**Published:** 2019-11-27

**Authors:** Chantal Brämer, Lisa Tünnermann, Alina Gonzalez Salcedo, Oscar-Werner Reif, Dörte Solle, Thomas Scheper, Sascha Beutel

**Affiliations:** 1 Institute of Technical Chemistry, Callinstraße 5, 30167 Hannover, Germanylisa.tuennermann@yahoo.com (L.T.); mehl@iftc.uni-hannover.de (A.G.S.); solle@iftc.uni-hannover.de (D.S.); scheper@iftc.uni-hannover.de (T.S.); 2 Sartorius Stedim Biotech, August-Spindler-Straße 11, 37079 Göttingen, Germany; oscar.reif@sartorius-stedim.com

**Keywords:** monoclonal antibody, membrane adsorber, protein A chromatography, periodic counter-current chromatography

## Abstract

Monoclonal antibodies are conquering the biopharmaceutical market because they can be used to treat a variety of diseases. Therefore, it is very important to establish robust and optimized processes for their production. In this article, the first step of chromatography (Protein A chromatography) in monoclonal antibody purification was optimized with a focus on the critical elution step. Therefore, different buffers (citrate, glycine, acetate) were tested for chromatographic performance and product quality. Membrane chromatography was evaluated because it promises high throughputs and short cycle times. The membrane adsorber Sartobind^®^ Protein A 2 mL was used to accelerate the purification procedure and was further used to perform a continuous chromatographic run with a four-membrane adsorber-periodic counter-current chromatography (4MA-PCCC) system. It was found that citrate buffer at pH 3.5 and 0.15 M NaCl enabled the highest recovery of >95% and lowest total aggregate content of 0.26%. In the continuous process, the capacity utilization of the membrane adsorber was increased by 20%.

## 1. Introduction

Monoclonal antibodies (mAb) “deliver considerable medical benefits” [1]. This is reflected in the increase in the number of available drugs based on monoclonal antibodies. In 2016, seven mAbs were approved in the US or EU and by the end of 2018, a further 12 mAbs had been approved in the US or EU for the treatment of e.g., cancer, transplant patients, autoimmune diseases and others [2,3].

Antibodies are a central component of the human immune system and are produced by activated B-cells as an immune response to the intrusion of antigens. They belong to the immunoglobulin family and are divided into five immunoglobulin classes (IgG, IgA, IgM, IgD and IgE), which are also subdivided into subgroups (e.g., IgG1, IgG2, IgG3, IgG4). Antibodies are glycoproteins and consist of four polypeptide chains, two heavy chains (50–60 kDa) and two light chains (23–25 kDa). The two identical heavy chains are connected by two disulfide bridges and each is linked by a disulfide bridge to one of the light chains. The light chains (LC) and heavy chains (HC) have a variable region (V-region) at one end that serves as a binding site for the antigen [4,5,6]. Monoclonal antibodies bind specifically to a defined epitope of an antigen and thus trigger the immune defense system; this has attracted interest in their medicinal applications [4]. The application of monoclonal antibodies in human medicine ranges from the treatment of allergies, asthma, multiple sclerosis, in the fight against various types of cancer and to their use in transplant patients [7,8,9].

**Figure 1:**
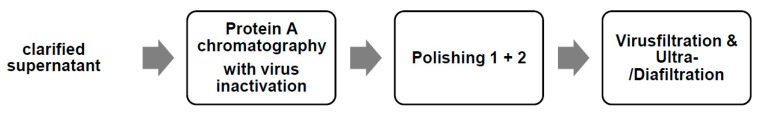
Process steps in the downstream processing of a monoclonal antibody (summarized and modified from [12]).

The production process for monoclonal antibodies is divided into production (upstream processing) and purification (downstream processing). The efficiency of the upstream process has increased significantly over the past few decades. The hybridoma technique has provided the basis for the targeted and reproducible amplification of monoclonal antibodies, so that now it is possible to produce monoclonal antibodies in recombinant cells on a large scale [6,7,10]. Production is often performed in Chinese Hamster Ovary (CHO) cells, whereby the monoclonal antibody is correctly glycosylated and secreted into the medium. Not only is the cultivation crucial for the production, but the following steps in the process must also be carefully considered. Subsequent downstream processing (DSP) must be of a high standard to meet all regulatory requirements and ensure clinical efficacy [11]. With increasing product titers in cultivation, the upstream processing (USP) costs do not increase significantly, while process costs in downstream processing increase in proportion to the quantity of the product to be purified. Therefore, with improved production, production costs shift to the DSP and can account for 50–80% [12] of total process costs [13,14,15,16]. DSP can be divided into a number of steps (shown in Figure 1), which should lead to a highly purified and effective drug.

After cultivation, the cells are separated from the supernatant (by e.g., centrifugation or depth filtration [17]); this is the last operation in the USP. Obtaining the clarified supernatant legally divides the entire process into the cell-containing process steps (USP) and the subsequent cell-free process steps (DSP). The first step in the chromatography is the capture step using Protein A chromatography in combination with virus inactivation. In this step, the monoclonal antibody should be isolated and concentrated from the supernatant, and the contaminants or impurities (DNA, host cell proteins and cell culture medium components) should be eliminated. The polishing steps (e.g., cation exchange chromatography, hydrophobic interaction chromatography, anion exchange chromatography) are then performed to remove the last impurities and achieve the final purity of the product. Ultrafiltration and diafiltration are used to obtain suitable buffer and formulation conditions [18,19].

These steps should be optimized in order to reduce the process costs for downstream processing. The most expensive step is the Protein A chromatography step; thus it offers a good starting point. This affinity chromatography method is based on the interaction of the monoclonal antibody with immobilized Protein A. The binding is primarily formed by hydrophobic interactions, but also hydrogen bonds and ionic interactions have an influence on the interaction [20,21]. The Protein A ligand was originally derived from the bacterium *Staphylococcus aureus* and serves as a binding site for IgG class antibodies in the cell wall. Protein A binds the antibody at the fragment crystallizable (Fc) region of the heavy chain [20,22,23]. Depending on the subclass of the antibody, the binding between the antibody and Protein A takes place in a pH range of 6–9 and can be influenced by the salt concentration in the binding buffer [5,20,24]. To release the binding, an elution buffer with a low pH between pH 2.5 and pH 4.5 is selected, taking into account that a low pH may affect the functionality and stability of the antibody and it may also support aggregate formation, which can lead to problems in further processing or in drug safety [18,20,22,25,26]. Since the elution of the antibody takes place at a low pH value, this is also used for virus inactivation. The eluate should be incubated for 30–120 min at a pH value lower than pH 3.8 in order to inactivate retroviruses [22,27].

The selection of a suitable elution buffer and the parameters for elution is particularly important for effective Protein A chromatography and good product quality. The elution can be optimized by additives [20,28,29] or the use of salts to prevent e.g., ionic interaction and thus increase the pH value during elution [20,24]. The addition of small amounts of salt can also have a positive effect on the stability of the antibody. Different buffer systems have already been tested for the purification of IgG_1_ antibodies with Protein A chromatography (citrate and acetate buffer) [30,31,32], whereas Müller and Vajda [32] observed better results with acetate buffer in regard to recovery. All authors observed that increasing pH had a negative effect on the recovery of the mAb. At pH 2.8–3.3, the recovery rate was higher than 90% whereas at pH 3.8 the recovery rate decreased to under 50%. Salt showed a negative effect on the elution in the work of Gagnon et al. [30]. Further, elution buffers were tested in regard to aggregate formation [32,33]: in the concentration range from 0–1.5 M NaCl, a negative effect of salt was observed and aggregation was induced while the pH value of the buffer also influenced the aggregate formation. Müller and Vajda [32] found about 1% aggregate in the range of pH 3–4. Singla et al. [34] investigated the aggregation kinetics, taking into account the pH, temperature, salt concentration (NaCl) and buffer species. They evaluated citrate, glycine and acetate buffer at pH 3.0 and found that these factors influenced aggregation in the following order with decreasing effect: pH, temperature, salt concentration and buffer species. At pH 3.0, citrate buffer induced the highest aggregation even without the addition of salt.

To address the issue that the process costs increase proportionally with the product titers in downstream processing, new optimization approaches were considered in this paper. One alternative is the use of other stationary phases such as disposable membrane adsorbers. They offer several advantages over conventional columns, e.g., higher throughputs and therefore shorter cycle times, an increase in productivity as well as easy up- and downscale of production, especially in the purification of low-concentrate products [35,36,37,38,39]. Some application examples of mAb purification with membrane adsorbers are summarized in Table 1.

In addition, continuous chromatography processes promise a further increase in productivity [45] and are therefore increasingly used in mAb processing [46,47]. It has been shown that continuous chromatography overcomes the problems of batch chromatography, whereby the capacity utilization of the stationary phase is significantly increased. This reduces the required amount of stationary phase, and hence reduces the material costs in DSP. This is especially interesting for the costly Protein A chromatography step in mAb purification.

In this study, Protein A chromatography was performed with a design of experiments (DoE) based approach to optimize the critical elution step for mAb purification. Membrane adsorbers were utilized to evaluate the potential of alternatives to conventional column chromatography and to accelerate the purification process. The implementation of continuous membrane chromatography was performed to further increase the productivity of the process and to evaluate the use of continuous chromatography with membrane adsorbers.

## 2. Materials and Methods

### 2.1. Materials

In this study a monoclonal antibody of the IgG_1_-type was used, which was produced with CHO cells in a serum-free medium. The antibody has a molecular weight of 148 kDa and a pI (isoelectric point) value at pH 8.25.

All chemicals were bought from Carl-Roth (Karlsruhe, Germany).

### 2.2. Methods

#### 2.2.1. Cell Separation with Depth Filtration

The monoclonal antibody was produced in CHO cells and secreted into the cell culture medium. Since the antibody must be purified for later use, the cells and cell debris were first removed from the culture supernatant. For this first step, two-stage depth filtration (first step: Sartoclear^®^ DL90, Göttingen, Germany; second step: Sartoclear^®^ DL20, Göttingen, Germany) was performed. The clarified material contained 1.9 g/L monoclonal antibody.

#### 2.2.2. Purification of the Monoclonal Antibody

The development of the Protein A chromatography method was performed using the Sartobind^®^ Protein A 2 mL membrane adsorber from Sartorius Stedim Biotech (Göttingen, Germany) and the ÄKTA™ pure system with a fraction collector from GE Healthcare (Uppsala, Sweden).

A chromatography run consisted of different phases. First the membrane adsorber was equilibrated with buffer A (phosphate-buffered saline (PBS) buffer, pH 7.4), then the clarified supernatant was applied on to the membrane adsorber and all unbound substances were removed by a washing step with buffer A. Elution with buffer B (see Table 2) was then followed by a cleaning in place (CIP) step and regeneration of the membrane adsorber. The buffers were connected to the ÄKTA inlets as follows: Inlet A1: buffer A, Inlet A2: CIP buffer (50 mM NaOH, 1 M NaCl). The sample was introduced via the sample loop (maximum sample volume 10 mL), and the various elution buffers (buffer B) from the design of experiments (DoE) in Table 2 were connected via inlets B1 to B5. In this study, three buffers systems for the monoclonal antibody elution were tested: 0.1 M citrate buffer (pH 2.5–4 and 0–0.5 M NaCl), 0.1 M glycine buffer (pH 2.5–4 and 0–0.5 M NaCl) and 0.1 M acetate buffer (pH 3.5–4 and 0–0.5 M NaCl). In the chromatography experiments, recovery (in %) and peak height (in mAU) were defined as target parameters. The ÄKTA™ was cooled to 10 °C and operated at a flow rate of 5 mL/min during the experiments.

The DoE was performed with a 2-factor design (pH and NaCl concentration), whereas chromatography performance was evaluated by using the target parameters, peak height and recovery, and antibody stability was evaluated by the monomeric antibody amount and the aggregate content. The software, MODDE^®^ (Umetrics, Version 12, Sweden) was used for planning and evaluation of the experiments. The models were selected according to the suggestions of the software.

#### 2.2.3. Stability Experiments

In order to perform the stability experiments, 19.2 µL of the already purified mAb were pipetted into 0.25 mL of the various elution buffers to be incubated (for 1 h, 24 h and one week at 20 °C, 4 °C and −20 °C). A SEC-HPLC as described in Section 2.3.1, was then performed. The evaluation stability was investigated by determining the monomeric antibody amount (in %) and aggregate content (in µV*mL or %).

### 2.3. Analytical Methods

#### 2.3.1. SEC-HPLC

Monoclonal antibody, fragment and aggregate concentration was determined using a commercial HPLC system operated with the column Yarra^TM^ 3 µm SEC-3000 (Phenomenex, Torrance, CA, USA). The samples for the SEC-HPLC were diluted as required, so that a concentration of about 0.5 g/L was obtained. The samples were then filtered (0.2 µm) and cooled in an autosampler (10 °C). The system was operated with a flow rate of 1 mL/min and a pressure of 112 bar. A total of 5 µL from each sample was added to the column (oven temperature 25 °C) and the measurement was carried out for 20 min. A combination of 100 mM Na_3_PO_4_ buffer and 100 mM Na_2_SO_4_ at pH 6.6 was used as the mobile phase.

#### 2.3.2. SDS-PAGE

For qualitative analysis of the monoclonal antibody, SDS-PAGE was carried out using a collecting gel with 6% acrylamide and a separation gel with 10% acrylamide. The samples were mixed with Lämmli buffer in the ratio 1:1 and boiled for 10 min at 95 °C. To run the gel, 1–10 µl of the sample as well as 5 µl of the Marker (PageRuler™ Unstained Protein Ladder, Thermo Scientific™, Waltham, MA, USA) were applied. For the separation, a voltage of 100 V was first applied to the loaded gel for 15 min, then the voltage was increased to 150 V for a further 60 min. For visualization, Coomassie staining [48] and silver staining [49] was used.

#### 2.3.3. Intrinsic Protein Fluorescence

To study the folding of the monoclonal antibody, the intrinsic protein fluorescence was measured with Tycho NT.6 (NanoTemper Technologies, Munich, Germany). For the measurement, 10 µL sample were used. During the measurement, the samples are thermally denatured by a temperature gradient and the fluorescence is detected. Due to the progressive denaturation, a slow unfolding of the proteins takes place whereby the amino acids, tryptophan and tyrosine, which fluoresce when excited with UV light, are increasingly exposed to the medium. As a result, the intrinsic protein fluorescence increases. A high value of intrinsic fluorescence indicates increased unfolding. For a good evaluation of the results obtained, they should be compared with a reference in order to evaluate whether the structure of the sample also corresponds to the desired product. The ratio of light absorption at a wavelength of 350 nm and 330 nm provides information on the quality of the protein [50].

### 2.4. Equipment

#### 2.4.1. Membrane Adsorber

For this study the Sartobind^®^ A membrane adsorber was used. The membrane adsorber consists of 20 layers (4 mm bed height) of stabilized reinforced cellulose (pore size 0.45 µm) and has a nominal adsorption area of 100 cm^2^, a bed volume of 2 mL and a ligand density of 1.5 mg/mL recombinant Protein A. Information was taken from the manufacturer’s user manual and the data sheet for the membrane adsorber. The dynamic binding capacity was determined as 5.9 mg/mL and the static binding capacity was 7.5 mg/mL.

#### 2.4.2. Chromatographic System

**Figure A1:**
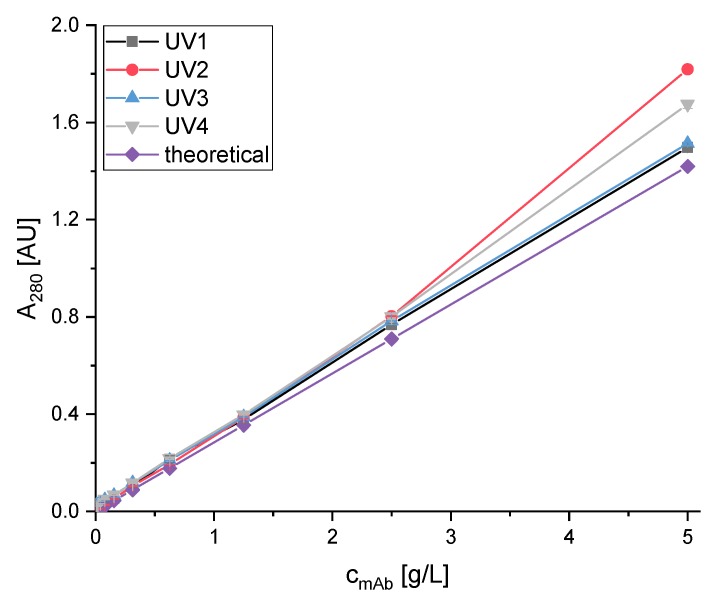
Calibration data of the mAb at the 4MA-PCCC system with a 2 mm flow through cuvette. Purified mAb solution was measured in the UV cuvettes. Small deviations occurred due to the use of optical fibers. Limit of detection (LOD) and limit of quantification (LOQ) were determined individually and the averages were calculated: LOD = 0.009 g/L and LOQ = 0.029 g/L.

The commercial system, ÄKTA™ pure (GE Healthcare) was used in this study. For continuous purification, a self-established chromatographic system with four membrane adsorbers (4MA-PCCC, Hanover, Germany) was used. The system was first described in [51] for use with three membrane adsorbers. A fourth chromatography unit with a measurement system was added to the system to provide more flexibility and functionality, and in particular, the implementation of the interconnected wash [51,52,53] to recover more product. The system is equipped with flow-through cuvettes with a 2 mm pathlength. A calibration curve was recorded with purified mAb and can be seen in Appendix A, Figure A1.

The continuous run was operated with a feed rate of 1.5 mL/min and a flow rate of 5 mL/min for buffer A and B. Switching conditions (SC1 and SC2) were set to 10 and 70% product breakthrough [54].

## 3. Results

### 3.1. Optimization of Chromatography Buffers

In order to achieve the best purification results, binding and elution buffers were tested and optimized for the membrane adsorber, Sartobind^®^ Protein A 2 ml. PBS at a neutral pH is often used as a standard binding buffer. Therefore, PBS binding buffer was tested in the pH range of 6–8 (see Appendix A). Here, no significant difference in chromatography performance was observed, hence standard PBS with pH 7.4 was chosen for monoclonal antibody binding.

The elution in Protein A chromatography is much more critical as a low pH value may lead to aggregation and denaturation of the antibody [32,33]. Therefore, the elution buffer was investigated in more detail. Above all, a compromise must be found between the performance and stability of the antibody.

#### 3.1.1. Screening of the Elution Buffers with Stability Testing

**Figure 2:**
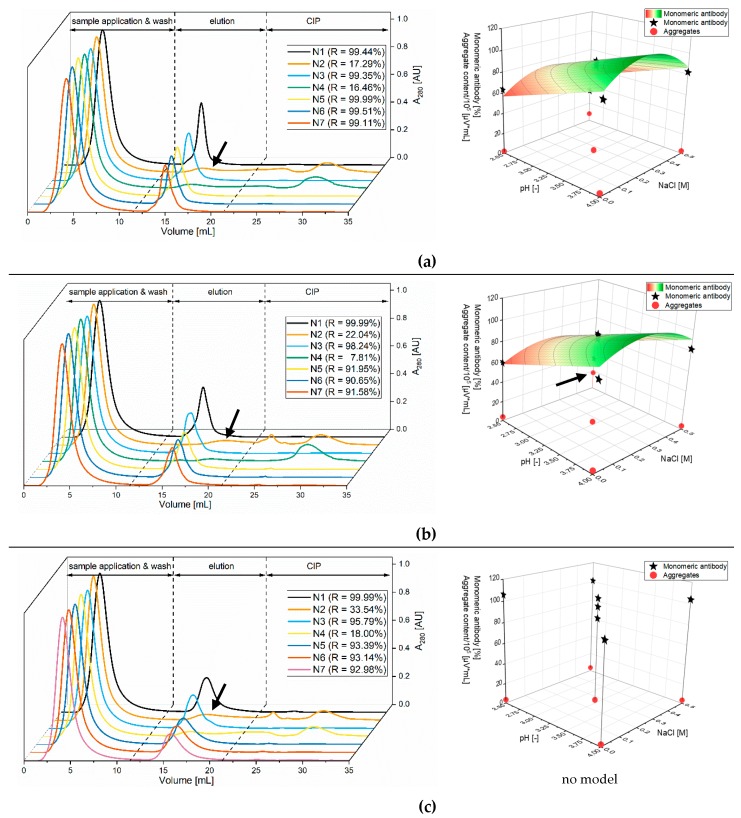
Chromatography (left), aggregation and stability (right) results of the DoE screening experiments with three elution buffers (**a**): citrate buffer pH 2.5–4, 0–0.5 M NaCl; (**b**): glycine buffer pH 2.5–4, 0–0.5M NaCl; (**c**): acetate buffer pH 3.5–4, 0–0.5 M NaCl). Aggregation and stability were measured after one day at 4 °C. Citrate buffer showed the best performance during chromatography runs as peaks were high and sharp (R = Recovery). The worst runs are marked with arrows. The aggregation was low for acetate buffer and increased at low pH and with salt (citrate and glycine buffer). The highest aggregation was measured with glycine buffer and is marked with an arrow. Stability experiments showed a clear trend: with increasing pH, the stability increases for citrate and glycine buffer. The models are shown with a surface diagram and experimental data was added.

**Figure A2:**
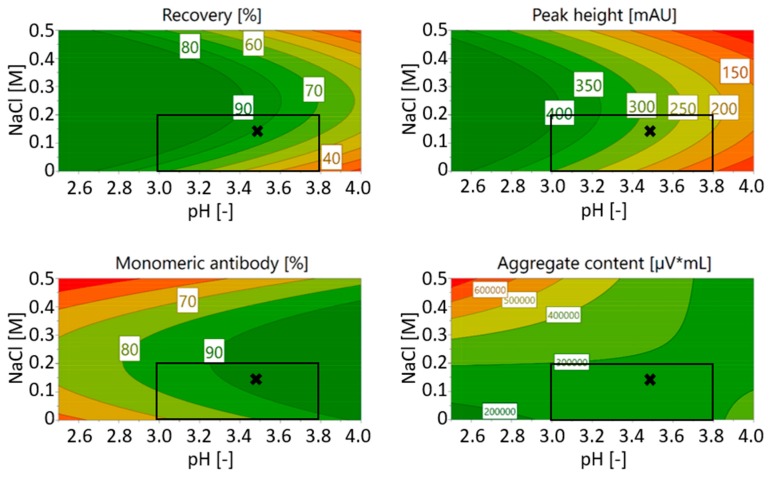
Results of the citrate buffer screening with chromatography experiments (Recovery and Peak height) and stability testing (Monomeric antibody and Aggregate content). The range for the next optimization step is marked with a box as well as the final operation point with a “×”.

In the first experimental series, a DoE screening with seven experiments for each buffer variant was performed (see Table 2). The results are shown in Figure 2 and are a summary of the results of the chromatography and stability experiments (not all data is shown, see Appendix A, Figure A2 for citrate buffer results).

A clear trend can be seen in the chromatography experiments. Citrate buffer provided the best results regarding recovery and peak height. The results for glycine buffers were comparable with citrate, but the peaks are flatter. Acetate buffer gave the worst results, although it should be noted that a smaller pH range was tested. The same trend can be observed for all three buffers: the lower the pH value, the higher the recovery and the peak height. The salt concentration, on the other hand, had only a minor influence. These observed trends correspond to the results of Mazzer et al. [25] and Müller and Vajda. However, they contradict the high influence of the salt content described by Gangnon et al. [30] and Bickel et al., at least in the ranges tested here.

In the stability tests (Figure 2, surface diagram) citrate and glycine buffer provided comparable results regarding the amount of intact monoclonal antibody. The effect was opposite to the chromatography results: the higher the pH value, the higher the proportion of intact antibody. The salt concentration had hardly any influence here but can have a non-linear effect depending on the buffer used, in the investigated range of 0–0.5 M NaCl. For the acetate buffer, the results showed no influence of pH and salt in the tested range.

In addition to chromatographic performance and the amount of intact antibody after incubation in elution buffer, aggregate formation was investigated. Figure 2 shows the results after incubation for 1 d at 4 °C. The greatest aggregation effects can be seen with the glycine buffer at low pH and high salt concentration. Similar tendencies were observed for citrate buffers, however less aggregates were formed. The aggregate formation for acetate in the pH range was also comparable to the other buffers. Singla et al. [34] also compared these three buffer species at a fixed pH of 3.0. Their results showed that at pH 3.0 citrate buffer induced the highest aggregate formation. In this work, the NaCl concentration was higher and up to 0.5 M NaCl compared to the 0.1 M NaCl used by Singla et al. This corresponds to the previously described order of aggregate formation that pH and salt concentration have a higher influence than the buffer species. However, here acetate and citrate buffer gave better results in regard to aggregation than glycine. Considering that temperature also has a major effect on aggregation, it was measured after one week at −20 °C (freezing–thawing). Glycine aggregation increased by 30% and the acetate and citrate buffer was increased by 100%. 

Taking into account the previous results, the citrate buffer was selected for further optimization. The citrate buffer provided the best results for chromatography with low aggregate formation and stable antibody in the experimental area. As storing at low pH values is not usual, the aggregation after one week is important but is not performed in a bioprocess.

#### 3.1.2. Optimization of Citrate Buffer

**Figure 3:**
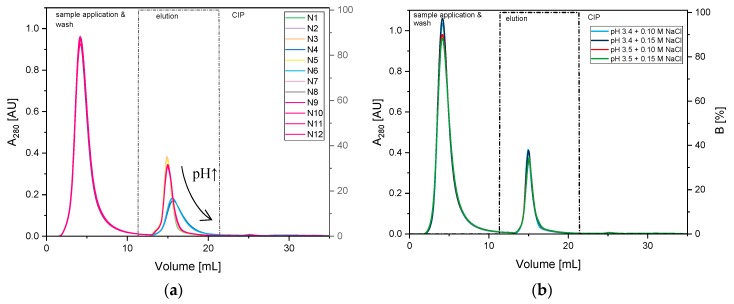
Optimization of Protein A chromatography elution with citrate buffer (**a**) and robustness testing (**b**). By increasing the pH, the performance of the chromatography decreased and the peaks became flatter. During the robustness testing, the result of the chromatography was not influenced by small fluctuations in the buffer system.

**Figure A3:**
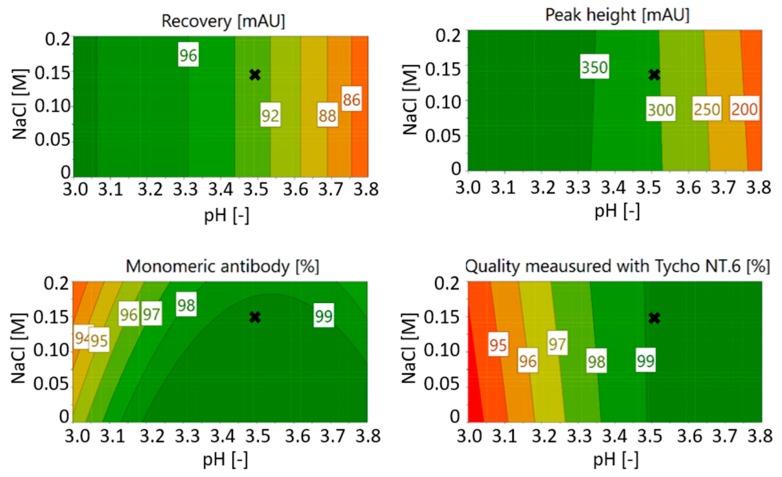
Results of the citrate buffer optimization with chromatography experiments (Recovery and Peak height) and stability testing (Monomeric antibody and Quality measured with Tycho NT.6). The final operation point is marked with a “×”.

To optimize the citrate buffer, the test range was limited to pH 3–3.8 and 0–0.2 M NaCl (see Appendix A, Figure A2). Eleven experiments (see Table 2) were carried out according to a DoE experiment plan. Chromatography runs were performed according to the experimental plan (Figure 3a). For the stability experiments the incubation time was set to 60 min to imitate virus inactivation conditions. For the chromatography experiments, the same tendencies were observed as in screening: the lower the pH value, the higher the recovery and the peak height. Almost exclusively the pH-value influences the performance. However, a contrary effect can again be seen in the stability results measured with SEC-HPLC and Tycho NT.6 (see Appendix A, Figure A3). A low concentration of salt had a positive effect on the stability and prevents aggregation. Therefore, pH 3.5 and 0.15 M NaCl were chosen for the purification and the optimization and robustness were checked (Figure 3b). Under the selected conditions, the recovery of the monoclonal antibody after the capture step was >95%. After purification with the membrane adsorber Sartobind^®^ Protein A 2 mL, the sample contained 0.26% aggregates, which is comparable to the results of Müller and Vajda [32]. Taking into account the results after freezing and thawing for one week, the aggregate content might further increase, therefore, immediate buffer exchange is mandatory. These low values could be due to the experimental set up, which considers not only the pH but also the salt (NaCl) concentration. Further, the use of membrane adsorbers leads to decreased cycle times.

### 3.2. Continuous Membrane Purification of the Monoclonal Antibody

Continuous membrane chromatography was used to increase the productivity of the purification step further with the optimized method from Section 3.1.2. The continuous chromatography method is based on the principle of periodic counter-current chromatography (PCCC), which is controlled by the UV signal of the membrane adsorber outlet. Therefore, the feed was diluted 1:3 to be in the linear measuring range (A_280_ < ~1.5 AU) with a mAb concentration of 0.6 g/L.

#### 3.2.1. Double Breakthrough Curve

**Figure 4:**
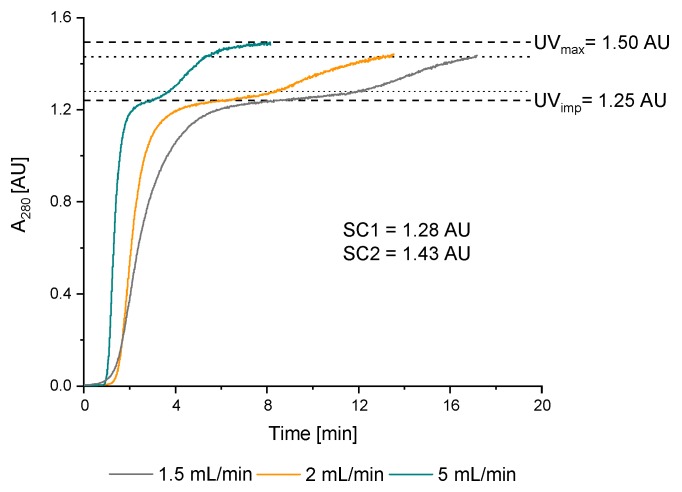
Determination of a suitable flow rate for loading of the Sartobind^®^ Protein A membrane adsorber for periodic counter-current chromatography (PCCC) operation. By increasing the flow rate, the double breakthrough curve got steeper. 1.5 mL/min was chosen as suitable flow rate.

In PCCC, up to two membrane adsorbers (MA) are loaded in series to capture the product breakthrough of the first MA on the second MA. The UV signal of the breakthrough curve serves as the dynamic control strategy for automation. This mode of operation allows loading of the MA close to the static binding capacity (available capacity of the MA in equilibrium) and thus results in a higher capacity utilization of the MA. To design the process, different breakthrough curves were recorded with the 4MA-PCCC (see Figure 4) to find the optimal feed rate and to determine the switching conditions for the dynamic process control with the UV signal. The feed rate of 1.5 mL/min was found to be suitable because the time for loading was equal to the time for regeneration, which is a criterion for PCCC [55]. 

The switching conditions were calculated as follows:

SC1 = 10%; ∆UV + UV_imp_ = 1.28 AU (1)

SC2 = 70%; ∆UV + UV_imp_ = 1.43 AU (2)

where ∆UV is the difference between the UV signal of the feed (UV_max_) and the UV signal of the impurity (here UV_imp_).

#### 3.2.2. PCCC Application

**Figure 5:**
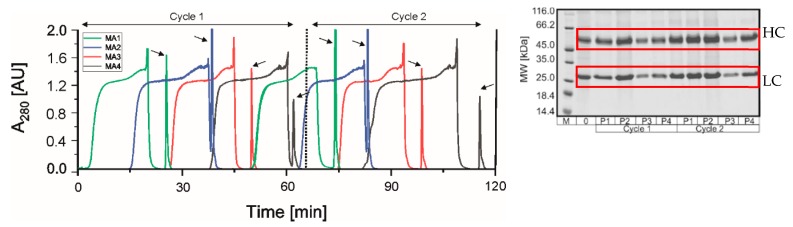
Chromatogram and reducing SDS-PAGE of two cycles with the 4MA-PCCC using Sartobind^®^ Protein A for the purification of a monoclonal antibody. Two PCCC cycles are shown in the chromatogram. A cycle consists of the loading, elution and regeneration of the four membrane adsorbers. The product peaks are marked with black arrows. Throughout the PCCC run, a trend was observed in that the peaks decrease during the cycles. The reason for this is the complex system setup.

The continuous run was performed with 160 mL feed containing 0.6 g/L mAb. The chromatogram can be seen in Figure 5. Two cycles are shown, which were performed in 2 h. The double breakthrough curves can be seen whereas the mAb elution is marked with arrows. The elution peaks depict concentrations between 2.5 and 5 g/L (see Appendix A, Figure A1). Due to the dynamic process control, each membrane adsorber was loaded until it was saturated. Slight differences occur due to the system setup. At the end of the loading a small peak can be seen, which was captured on a further membrane adsorber (interconnected wash [52,53]). A constant performance of the MA can be observed in the process, but a trend (MA1 to MA4) can be seen in each cycle. The elution peaks decrease because the self-built system has back suction from buffer B. In this system, the very complex circuit was realized with 37 valves and this resulted in a high dead volume, which causes the back suction. Nevertheless, the recovery after the purification was higher than 90% and only 600 mL buffer was consumed. Compared to the batch process, the capacity utilization of the MA was increased by 20% due to the loading principle of PCCC. Further, the optimized chromatography conditions showed a significant improvement in the PCCC compared to the non-optimized conditions (see Appendix A, Figure A3).

## 4. Discussion

This study presents the optimization of the critical Protein A purification step in monoclonal antibody downstream processing with a focus on the elution. Up until now, downstream processing has been a bottleneck in the production process, since high product titers must be processed in compliance with legal requirements. The Protein A step is critical because a low pH is used for product elution, which can lead to aggregation and thus to problems in quality, quantity, further processing and above all, in drug safety. It is therefore vital to optimize the downstream process. In this study, the elution was optimized with various buffers and the application of membrane adsorbers (Sartobind^®^ Protein A 2 mL) as an alternative to conventional column chromatography. Although the capacities were still lower than in column chromatography, the throughputs were significantly higher and the residence times were shorter. This is particularly interesting for mAb elution. For the IgG_1_ mAb, tested in this work, 0.1 M citrate buffer pH 3.5 + 0.15 M NaCl provided the best results in terms of MA chromatography performance, product stability and aggregation (tested with SEC-HPLC and Tycho NT.6). Furthermore, the development time of the process could be significantly reduced with membrane adsorbers. The optimized chromatography method was successfully transferred to a continuous chromatography system operated with four membrane adsorbers (4MA-PCCC).

The PCCC principle is very suitable for the purification of mAbs because the product concentration is quite high compared to other products [51,56]. The control via the UV signal using the switching conditions could be easily implemented. Due to increasing product titers > 5 g/L [57], this will improve even further. This is particularly useful in relation to the process analytical technology (PAT) initiative defined by the FDA [58]. The process can be monitored directly and the performance is directly visible. The process can be stopped before the performance decreases and the product quality no longer meets the legal requirements. This could increase the economics of the DSP, since better utilization of the MA used would increase productivity.

In conclusion, this work shows the structured optimization of a monoclonal antibody purification step. It is very important to control the quality of the antibody throughout the entire optimization process in order to ensure high product quality and to identify the critical steps. By using DoE, the number of experiments could be reduced and thus the optimization was very fast. The use of membrane adsorbers has the advantage that the process is easily scalable and the disposable MA can be disposed of after use. Furthermore, the transfer of the batch to continuous operation mode was described, and this is very relevant to current developments in the biotechnological industry.

## Figures and Tables

**Table 1:** Application examples of membrane chromatography for monoclonal antibody (mAb) purification.

Field of Application	Ref.
Affinity chromatography	[40]
Ion exchange chromatography	[40,41,42,43]
Hydrophobic interaction chromatography	[41,42]
Accelerated, Seamless Antibody Purification (ASAP)-continuous method (Protein A chromatography, cation and anion exchange chromatography)	[44]

**Table 2:** Design of experiments (DoE) screening for 0.1 M citrate-, 0,1 M glycine-buffer and 0.1M acetate, N5–N7 are the center points.

Screening ^1^								Optimization ^2^			
Citrate/Glycine Buffer				Acetate Buffer				Citrate Buffer			
Exp.	Order	pH	NaCl [M]	Exp.	Order	pH	NaCl [M]	Exp.	Order	pH	NaCl [M]
N1	1	2.5	0	N1	1	3.5	0	N1	4	3	0
N2	7	4	0	N2	7	4	0	N2	7	3.8	0
N3	2	2.5	0.5	N3	2	3.5	0.5	N3	2	3	0.2
N4	4	4	0.5	N4	4	4	0.5	N4	6	3.8	0.2
N5	5	3.25	0.25	N5	5	3.75	0.25	N5	10	3	0.1
N6	6	3.25	0.25	N6	6	3.75	0.25	N6	1	3.8	0.1
N7	3	3.25	0.25	N7	3	3.75	0.25	N7	3	3.4	0
								N8	11	3.4	0.2
								N9	5	3.4	0.1
								N10	8	3.4	0.1
								N11	9	3.4	0.1

^1^ Full Fac 2 level, interaction model, fitted with: PLS; ^2^ CCF, quadratic model, fitted with: PLS.
